# Forebrain corticosteroid receptors promote post-myocardial infarction depression and mortality

**DOI:** 10.1007/s00395-022-00951-6

**Published:** 2022-09-06

**Authors:** Bastian Bruns, Ricarda Daub, Thomas Schmitz, Maria Hamze-Sinno, Sebastian Spaich, Matthias Dewenter, Chrysovalandis Schwale, Peter Gass, Miriam Vogt, Hugo Katus, Wolfgang Herzog, Hans-Christoph Friederich, Norbert Frey, Jobst-Hendrik Schultz, Johannes Backs

**Affiliations:** 1 Institute of Experimental Cardiology, Heidelberg University, Heidelberg and DZHK (German Centre for Cardiovascular Research), Partner Site Heidelberg/Mannheim, Im Neuenheimer Feld 669, 69120 Heidelberg, Germany; 2 Department of General Internal Medicine and Psychosomatics, University of Heidelberg, Heidelberg, Germany; 3 Institute for Physiology and Pathophysiology, University of Heidelberg, Heidelberg, Germany; 4 Department of Cardiology, Angiology and Pneumology, University of Heidelberg, Heidelberg, Germany; 5 Central Institute of Mental Health, RG Animal Models in Psychiatry, Medical Faculty of Mannheim/University of Heidelberg, Mannheim, Germany; 6 Department of Cardiology and Angiology, Robert-Bosch-Krankenhaus, Stuttgart, Germany; 7 DZHK (German Centre for Cardiovascular Research), Partner Site, Heidelberg/Mannheim, Germany

**Keywords:** Mineralocorticoid receptor, Glucocorticoid receptor, Myocardial infarction, Limbic system, Heart failure, Depression

## Abstract

Myocardial infarction (MI) with subsequent depression is associated with increased cardiac mortality. Impaired central mineralocorticoid (MR) and glucocorticoid receptor (GR) equilibrium has been suggested as a key mechanism in the pathogenesis of human depression. Here, we investigate if deficient central MR/GR signaling is causative for a poor outcome after MI in mice. Mice with an inducible forebrain-specific MR/GR knockout (MR/GR-KO) underwent baseline and follow-up echocardiography every 2 weeks after MI or sham operation. Behavioral testing at 4 weeks confirmed significant depressive-like behavior and, strikingly, a higher mortality after MI, while cardiac function and myocardial damage remained unaffected. Telemetry revealed cardiac autonomic imbalance with marked bradycardia and ventricular tachycardia (VT) upon MI in MR/GR-KO. Mechanistically, we found a higher responsiveness to atropine, pointing to impaired parasympathetic tone of ‘depressive’ mice after MI. Serum corticosterone levels were increased but—in line with the higher vagal tone—plasma and cardiac catecholamines were decreased. MR/GR deficiency in the forebrain led to significant depressive-like behavior and a higher mortality after MI. This was accompanied by increased vagal tone, depleted catecholaminergic compensatory capacity and VTs. Thus, limbic MR/GR disequilibrium may contribute to the impaired outcome of depressive patients after MI and possibly explain the lack of anti-depressive treatment benefit.

**Supplementary Information:**

The online version contains supplementary material available at 10.1007/s00395-022-00951-6.

## Introduction

Depression following myocardial infarction (MI) presents an independent risk factor for mortality with an impact equivalent to Killip class and history of previous MI [15]. However, several forms of psychotherapy as well as antidepressant medication failed to reveal a beneficial effect on prognosis [56]. Even though a number of associations have been suggested clinically [30] and several mechanisms—e.g. vegetative imbalance, neurotrophic mediators, and cardiac apoptosis—have been implicated experimentally [17, 39, 47, 58], the etiology of increased mortality after MI in the presence of clinical depression remains largely unknown.

The ‘corticosteroid receptor (CR) hypothesis of depression’ postulates impaired central corticosteroid receptor signaling as a key molecular mechanism underlying the pathogenesis of human depression [28, 52]. In line with this concept, polymorphisms in human genes encoding the mineralocorticoid (MR) and glucocorticoid receptor (GR) were found to be associated with depression and altered hypothalamic–pituitary–adrenal (HPA) axis function [53]. Specifically, disruption of the limbic (forebrain) MR/GR equilibrium may be causal to dysregulation of the HPA-axis and subsequent impairment of behavioral adaptation to stress, resulting in depression after aversive events [38] such as MI. The MR and GR facilitate the actions of systemic corticosteroids, which display circadian activity and progress with exposure to stress [6]. While the MR is mostly expressed in limbic structures, especially in the hippocampus [33], and binds corticosterone with a tenfold higher affinity [3], with continuous occupation at basal levels of HPA-axis activity, the GR is expressed throughout the entire brain. High levels of circulating corticosterone, at circadian peak or due to stress, progressively saturate the GR [3]. Mechanistically, the forebrain MR has been implicated in setting the threshold at the onset of the activation of the stress response, featuring downstream activation of the HPA-axis and the sympathetic nervous system (SNS), whereas the role of the GR appears to be its termination [10]. Usage of mutant mice with an inducible knockout avoids the problem of confounding developmental adaptations. Cardiac effects of the vegetative nervous system are implemented by adrenal systemic catecholamine release and via local cardiac norepinephrine (NE) release based on tyrosine hydroxylase (TH)-driven production within adrenals and sympathetic ganglia. The later converge on the cervicothoracic ganglia, particularly the stellate ganglion. Regional NE reuptake is driven by the norepinephrine transporter (NET) [4].

The underlying mechanism of impaired outcome in the setting of cardiac disease with comorbid depression remains unknown. Here, we confirmed MR and GR double KO mice to be vulnerable to stress-induced depressive-like behavior, thereby establishing a brain-specific model for murine depression which cab used to study the consequences on the heart. Taking advantage of this model for the first time, we show that impaired forebrain CR signaling increases mortality after experimental MI possibly through vegetative imbalance.

## Methods

### Experimental animals

In this study, we used mice harboring a conditional knockout in the murine forebrain, based on combination of the Cre/loxP-system with a tamoxifen-inducible fusion protein, comprising Cre recombinase and the mutated ligand binding domain of the human estrogen receptor (CreERT2) under the control of the regulatory elements of the Ca2 + /calmodulin-dependent protein kinase IIα (CaMKIIα) gene (CaMKIIα-CreERT2 transgene). Conditional allels of MR [6] and GR [51] were generated before and kindly provided by S. Berger (DKFZ, Heidelberg, Germany). Mice homozygous for the conditional MR and GR alleles were bred to CaMKIIα-CreERT2 transgene [12]. Cre-positive knockouts (KO) and cre-negative controls (‘Ctrl.’, MR/GR double floxed) were treated with 1 mg tamoxifen twice a day for 5 consecutive days to induce mutagenesis as described before [6, 12, 55]. MR/GR-KO mice show a residual target gene expression of 30%. All mutant mice had been backcrossed to the C57BL/6 N background for at least ten generations. Genotypes were verified by CaMKIIα-CreERT2 polymerase chain reaction (PCR).

All animal experiments were performed according to institutional and governmental guidelines. This investigation followed the Principle of Laboratory Animal Care (NIH Publication No. 86–23, revised 1985) and was approved by the authorities of the Regierungspräsidium Karlsruhe (G-31/16, G-109/16, G-208/17). Every effort was made to minimize the number of animals used, and their suffering. Animals were held with access to food and water ad libitum at a 12 h day-and-night-rhythm at 21 °C and 50–60% humidity. Behavioral testing was conducted during the active phase of the animals.

### Experimental design

Male wildtype (‘Ctrl.’, cre-negative, GR/MR double-floxed) and knockout (KO, cre-positive, GR/MR double-floxed) mice were intraperitoneally injected with 1 mg of tamoxifen at the age of 9–12 weeks with a subsequent recovery period of 4 weeks. Afterward, baseline echocardiography was performed and myocardial infarction (MI) or SHAM operation were conducted. At 24 h blood was drawn to quantify infarct size via high-sensitive cardiac troponin T. Follow-up echocardiography was performed 24 h, 2-, 4-, and 6 weeks after MI. Behavioral testing was conducted in identical order at 4 weeks post-MI over 3 consecutive days (Openfield (d1), Tail-Suspension (d2), and Forced-Swim Test (d3)) by an experienced investigator blinded regarding the animals group affiliation. After each animal, the corresponding area / apparatus was cleaned with 70% ethanol. Animals were sacrificed after 24 h (cohort 1), 4 (cohort 2), or 6 weeks (cohort 3). Organ harvest 4 weeks after MI was conducted between 8 and 10 AM. Non-infarcted myocardium was used for further analysis. In a subgroup of cohort 3, telemetry transmitters were implanted 2 weeks after tamoxifen application with a consecutive 2-week recovery period followed by a 3-day baseline measurement with subsequent surgical induction of MI and a 6-week follow-up. To assess parasympathetic drive 1-, 3-, and 10 mg/kg bw atropine were injected once during baseline and once during follow-up measurements with a 2 h pause between trials.

### Myocardial infarction model

Permanent left anterior descending artery (LAD) ligation was used to induce myocardial infarction (MI). Mice were injected with buprenorphine 0.1 mg/kg bodyweight (bw) subcutaneously (s.c.) 30 min before operation for sufficient perioperative analgesia. Under anaesthesia with isoflurane (3 vol.%) mice were intubated and ventilated. Parasternal incision was followed by cleavage of the pectoral muscle. After thoracotomy in the third intercostal space proximal LAD ligation with an 8–0 nylon suture induced blanching of the distal myocardium verifying myocardial infarction. In sham-operated mice the myocardium was punctured without ligation. The operation lasted 20 min with subsequent postoperative analgesia with s.c. buprenorphine.

### Plasma/serum preparation, measurement of Troponin T and corticosterone

Retroorbital blood was collected from isoflurane anaesthetized mice using heparin-coated hematocrit capillaries 24 h after LAD or SHAM procedure and centrifuged at 14000 rpm for 20 min at 4 °C. Plasma samples were then stored at − 80 °C until further analysis. For quantification of infarct size, high-sensitive cardiac Troponin T (hs-TnT) was measured using an automated Cobas Troponin T hs STAT Elecsys (Roche) as described previously [59] at the Central Laboratory of the University Hospital Heidelberg (Department of Endocrinology and Clinical Chemistry). Also, at the corresponding end point of each experiment, trunc blood was collected after decapitation and rapidly centrifuged at 14,000 rpm for 20 min at 4 °C. Serum was separated and stored until further analysis at − 80 °C. Measurement of corticosterone was conducted in serum by radio-immunosorbent assay (RIA) as described before [41] at the Steroid Laboratory of the University Hospital Heidelberg (Department of Pharmacology).

### Echocardiography

Cardiac function was assessed by 2D echocardiography at baseline, after 24 h, 2-, 4-, and 6 weeks on conscious mice. Echocardiography was performed using a Visual Sonics Vevo® 2100 using a MX550D transducer. The investigator was blinded with respect to the treatment group. Mice were shaved and left ventricular parasternal short-axis views were obtained in M-mode imaging at the papillary muscle level. Three consecutive beats were used for measurements of left ventricular end-diastolic internal diameter (LVEDD) and left ventricular end-systolic internal diameter (LVESD). Fractional shortening (FS) was calculated as FS% = [(LVEDD—LVESD)/LVEDD] × 100%.

### In vivo telemetry recording in awake mice

Implantation of ECG transmitters (ETA-F10, DSI) was performed on anaesthetized mice (2% isoflurane). Transmitters were implanted subcutaneously with the negative electrode fixed to the right pectoralis fascia and the positive electrode 1 cm left to the xiphoid. Mice received buprenorphine 0.1 mg/KG s.c. 30 min before and 8 h after the operation. Recordings were started after a recovery time of 2 weeks. Each mouse was monitored continuously. Recording and analysis parameters were set according to the manufacturer’s instructions using Ponemah 5.2 software (DSI).

### Openfield test

Locomotor testing was conducted 4 weeks after MI in a square, grey openfield arena of 50 × 50cm^2^ with 25 lx illumination from above. Mice were placed individually into the arena and tracked for 10 min. by a digital camera from above. Tracking and evaluation were performed with Limelight 4.1 (Actimetrics). Evaluated parameters included mean distance to center (cm), total distance moved (cm) and mean speed (cm/s).

### Tail-suspension test

Briefly, experimental animals were isolated and suspended 25 cm above the floor 4 weeks after MI by the tip of the tail with tracking of immobility by a digital camera for 6 min. Mice were considered immobile only when they hung completely motionless. Image acquisition and evaluation were conducted with FreezeFrame 4.0 (Actimetrics).

### Porsolt-forced-swim test

Mice were placed individually into a glass cylinder (23 cm height, 17 cm diameter), filled with water at 21 °C up to a height of 12 cm and monitored for 6 min by a digital camera from the side. The first 2 min were accounted for as an acclimatization period with evaluation of the latter 4 min. Immobility was defined as floating with only movements, necessary to stay above water. Struggling/swimming was defined as time spent in active movement. Immobility was evaluated with FreezeFrame 4.0 (Actimetrics). The water was switched after each animal. Afterward, mice were placed in front of a heating lamp to support quick recovery of body temperature.

### Measurement of plasma and left ventricular tissue catecholamines

Catecholamine levels were quantified in plasma and left ventricular tissue. Cardiac tissue was weighted and subsequently homogenized in an ice-cold solution (0.01 M HCl, 1 mM EDTA, 4 mM Sodium disulfide). Whole blood was centrifuged at 14,000 rpm for 20 min. at 4 °C and diluted 1:40 with the same ice-cold solution. Measurements were performed in each sample using high-performance liquid chromatography (HPLC) coupled with electrochemical detection (potential 0.48–0.6 V, range 20 nA) at the Central Laboratory of the University Hospital Heidelberg (Department of Endocrinology and Clinical Chemistry). Calibration was conducted according to an internal standard (dihydroxybenzylamine, Chromosystems). After 3xwashing of the samples with washing buffer (3 × 1 ml, Chromosystems) and centrifugation at 2 × 2000 U/min, and finally 1 × 4000 U/min, 120 µl elution buffer were added for 5 min with subsequent additional centrifugation at 2000 U/min and addition of 20 µl 1 M HCl before quantification. Volumes of 50 µl were automatically injected. The flow rate was 1 ml/min. The detection limit for dopamine was 60 ng/l (391.8 pmol/l), for norepinephrine 50 ng/l (295.5 pmol/l), and for epinephrine 50 ng/l (273 pmol/l). Results were calculated in pmol/l for plasma catecholamines and pg/mg for tissue levels.

### Protein extraction and Western blot

Left ventricular (LV) tissue was homogenized in Tris buffer (20 mM, pH = 7.4, Carl Roth), supplemented with phenylmethylsulfonyl fluoride (1:100, Carl Roth), protease inhibitor cocktail (1:50, Sigma), and a phosphatase inhibitor cocktail (1:100, Sigma) at 2 °C. After incubation with 25 μl Nonidet P-40 10% (Fluka Analytical) probes were centrifuged for 10 min at 14000 rpm at 4 °C. Supernatants were snap frozen in liquid nitrogen and stored at − 80 °C. BSA standards and samples were measured according to the bicinchoninic acid (BCA) method in duplicates by photometric absorption at 562 nm. Lysed protein extracts were diluted with GST buffer and brought to equal concentration levels. For protein denaturation, 1:6 Laemmli buffer (415 mM SDS, 0.1 mM Bromophenol blue, 0.5 M TRIS pH 6.8, 50% Glycerol, 600 mM DTT + 1:20 ß-Mercaptoethanol) was added and probes were incubated at 95 °C for 5 min. Electrophoretic separation was conducted through a 15% SDS–polyacrylamide gel. Laddered proteins were transferred to a polyvinylidene difluoride (PVDF, Merck Millipore) membrane. After blocking membranes were incubated overnight at 4 °C with the respective primary antibody. After washing, membranes were incubated at room temperature with the respective horseradish-peroxidase (HRP)-conjugated-secondary antibody. After washing, visualization was conducted on a chemiluminescence imager (Fusion FX). Antibodies for immunoblotting were: PLN (Badrilla, A010-14, 1:5000), PLN-Ser16 (Badrilla, A010-12, 1:5000), PLN-Thr17 (Badrilla, A010-13, 1:5000). Densitometry was evaluated by the NIH software Image J [46].

### RNA extraction and quantitative PCR

Total RNA was isolated from homogenized left ventricular-, prefrontal cortex-, hippocampal-, hypothalamic-, stellate-, and adrenal tissue using TRIzol (Invitrogen). Samples were incubated for 3 min in after adding chloroform at room temperature, and centrifuged for 15 min at 12,000 rpm at 2 °C. The RNA-containing aqueous phase was gently mixed with 500 μl of a prepared solution composed of glycogen (Invitrogen) and isopropanol (Sigma). Precipitation was carried out overnight at − 20 °C. Next, samples were centrifuged, and pellets washed with 70% Ethanol/30% DEPC-H_2_O. After drying, pellets were solved in DEPC-H_2_O and incubated at 68 °C at 800 rpm. To avoid RNA digestion all relevant surfaces were treated with an RNAase decontamination agent (Steinbrenner Laborsysteme) during RNA isolation. RNA concentration was measured by photometry and simple purity was verified (OD_260_/OD_280_ > 1.8). Complementary DNA (cDNA) synthesis of 500 ng RNA was carried out using the Super-Script first-strand synthesis system for RT-PCR (Invitrogen). After RNA denaturation and complementary apposition of bases, cDNA mix reagents (4 µl reaction buffer, 2 µl 10 mM dNTP mix, 1 µl RiboLock RI, 2 µl M-MulV RT; all Thermo Fisher Scientific) were added and probes were incubated at room temperature for 5 min and reversely transcribed at 37 °C for 60 min with subsequent reaction termination at 70 °C for 5 min. 5 µl of each cDNA probe were used for standard preparation, the rest was diluted 1:5 with DEPC-H2O and brought to a total RNA—concentration of 10 ng/µl. Quantitative real-time PCR was performed using the Universal Probe Library (Roche) with the TaqMan Universal PCR Mastermix (Applied Biosystems) and detection on a 7500 Fast Cycler (Applied Biosystems). For standard preparation, 5 µl of all non-diluted cDNA samples were combined to a concentration of 30 ng/µl with DEPC-H_2_O, followed by serial 1:2 dilution until 1.125 ng/µl. The rest of the cDNA probes were diluted 1:5 with DEPC-H_2_O. For quantitative real-time PCR, 2 µl of each standard or 2 µl of diluted cDNA samples were mixed with 8 µl Mastermix (5 µl Tagman, 0.4 µl forward primer, 0.4 µl reverse primer, 0.1 µl Universal Probe, 3.1 µl DEPC H_2_O). Real-time quantification was performed with fluorescence resonance energy transfer (FRET-) probes in duplicates. Evaluation was performed with the corresponding analysis software (Applied Biosystems), by reference to the standard curve. RNA concentration of each sample was normalized to gapdh for final mRNA quantification. The following primer sequences were used: gapdh 5′-ccttgagatcaacacgtaccag-3′ and 5′-cgcctgtacactccaccac-3′, nppb 5′-gtctggccggacactcag-3′ and 5′-tgcactggtgtcttcaacaac-3′, MR 5′-cctggcagcgaaacagat-3′ and 5′-tcctcgagaggcaagttttt-3, GR 5′-caaagattgcaggtatcctatgaa-3′ and 5′-cttggctcttcagaccttcc-3′, TH 5′-cccaagggcttcagaagag-3′ and 5′-gggcatcctcgatgagact-3′.

### Statistical analysis

The results are expressed as the mean ± SEM. Normal distribution was verified by the Kolmogorov–Smirnov test. Statistical analysis included one-way ANOVA or Kruskal–Wallis test followed by Bonferroni or Dunn’s post hoc test, respectively. Student’s *t* or Mann–Whitney *U* test were used when appropriate. Kaplan–Meier survival analysis was performed using the log-rank test. A *p* < 0.05 was considered statistically significant.

## Results

### Ablation of the forebrain MR/GR per se does not impact corticosterone or catecholamines

Inducible CaMKIIα-CreERT2-driven ablation of MR and GR significantly blunted MR and GR mRNA expression in structures of the murine forebrain, including prefrontal cortex (Suppl. Figure 1A-B) and hippocampus (Suppl. Figure 1C-D), while not affecting expression in hypothalamus (Suppl. Figure 1E-F) as well as in the left ventricle of the heart (Suppl. Figure 1G-H).

To exclude an impact of the MR/GR-KO per se at baseline in “unstressed” mice without myocardial infarction or sham operation, serum levels of corticosterone, plasma-, and cardiac catecholamines were compared. We observed no marked differences in corticosterone (Supplementary Fig. 2A), epinephrine (Supplementary Fig. 2C), and norepinephrine (Supplementary Fig. 2E) as well as left ventricular cardiac epinephrine (Supplementary Fig. 2B) or norepinephrine (Supplementary Fig. 2D) when compared to controls (Ctrl.).

### Ablation of the forebrain MR/GR exacerbates mortality and depressive-like behavior upon MI

**Fig. 1:**
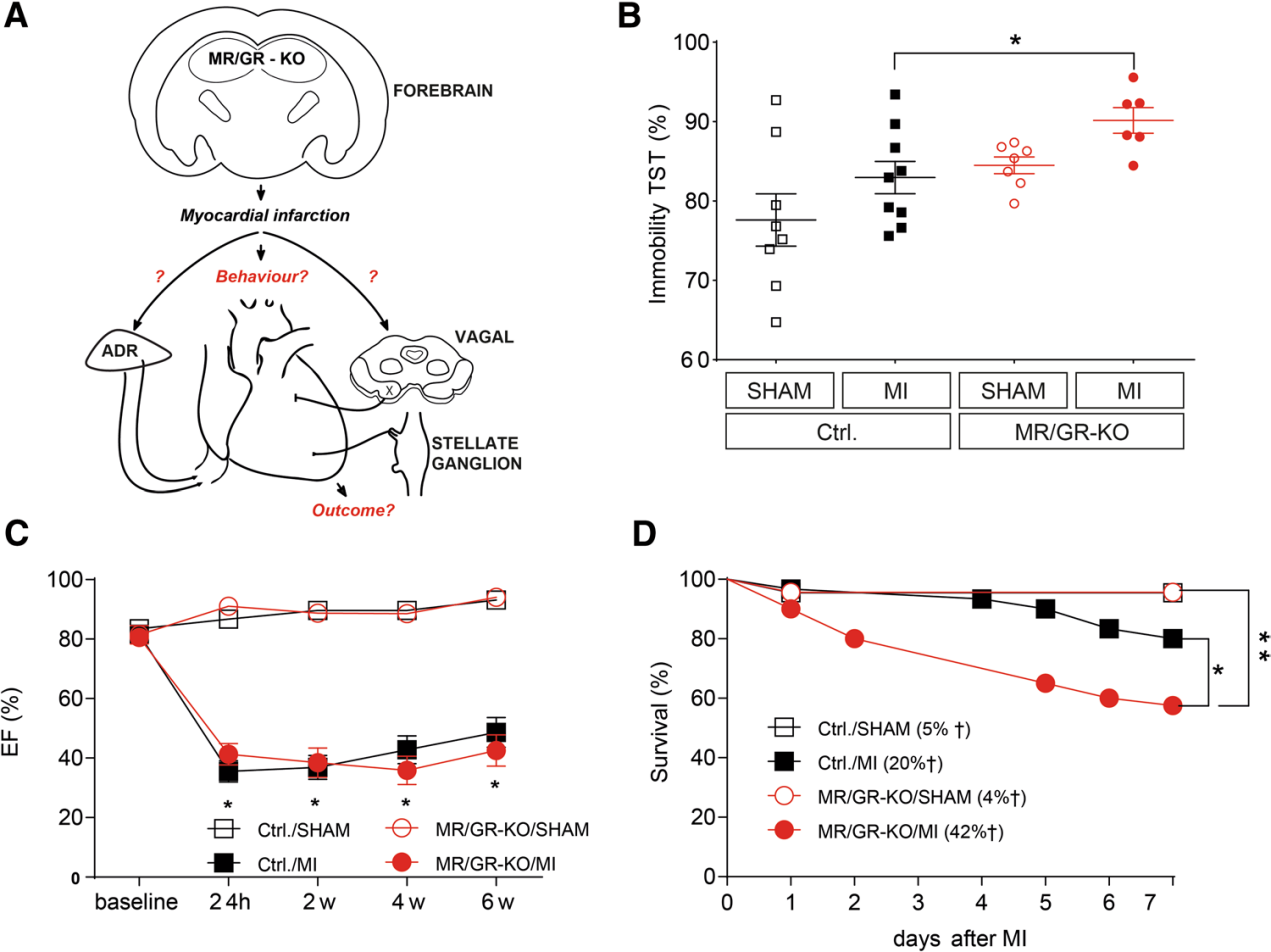
Ablation of the forebrain MR/GR exacerbates depressive-like behavior and mortality. Cartoon depicting study design (**A**): the impact of myocardial infarction on mice with a forebrain-specific corticosteroid receptor knockout (MR/GR-KO) was investigated regarding adrenal (ADR, corticosterone (CS), epinephrine (E)/norepinephrine (NE)) as well as cardiac vegetative (vagal (X), stellate ganglion (SY)) impact. In the Tail-Suspension test MR/GR KO displayed significant immobility when compared to control mice (Ctrl.) 4 weeks after MI (*n* = 6–8/group) (**B**). Left ventricular ejection fraction (EF) was reduced 24 h, 2 weeks, 4 weeks, and 6 weeks after MI with no difference between Ctrl. and KO. Also, KO mice show no cardiac phenotype at baseline when compared to Ctrl. mice (*n* = 11–18/group) (**C**). MR/GR KO mice display significantly increased mortality early after MI (*n* = 23–40/group) (**D**). Data are presented as mean ± SEM. *p < 0.05, **p < 0.01 by ANOVA or Log-rank test, respectively

Cardiac function was unaffected by the MR/GR-KO at baseline, and we observed a comparable degree of heart failure in Ctrl. and MR/GR-KO mice 24 h, 2 weeks, 4 weeks, and 6 weeks after MI (Fig. 1C) as well as similarly elevated high-sensitive cardiac troponin T (hs-TnT) at 24 h (Suppl. Figure 3A) with no difference between Ctrl. and MR/GR-KO mice. In line with these findings, the expression of brain-natriuretic peptide (Nppb) was elevated after MI in Ctrl. and KO mice with no significant differences between genotypes (Suppl. Figure 3B). We observed no impact on overall motility (total distance moved / mean speed, Suppl. Figure 4A, C) or anxiety-like behavior 4 weeks after MI (mean distance to center, Suppl. Figure 4B) in the Open field Test. But strikingly, MR/GR-KO mice revealed increased mortality early after MI (Fig. 1B). Autopsy was performed on each animal found dead to assess potential ventricular rupture. We did not observe ventricular rupture in MR/GR-KO mice. Also, MR/GR-KO mice displayed significant depressive-like behavior as quantified by the percentage of time spent immobile in the Tail-Suspension- (Fig. 1B) or Porsolt-Forced-Swim Test (Suppl. Figure 4D) 4 weeks after MI.

### Ablation of the forebrain MR/GR blunts sympathetic activity and facilitates VTs after MI

**Fig. 2:**
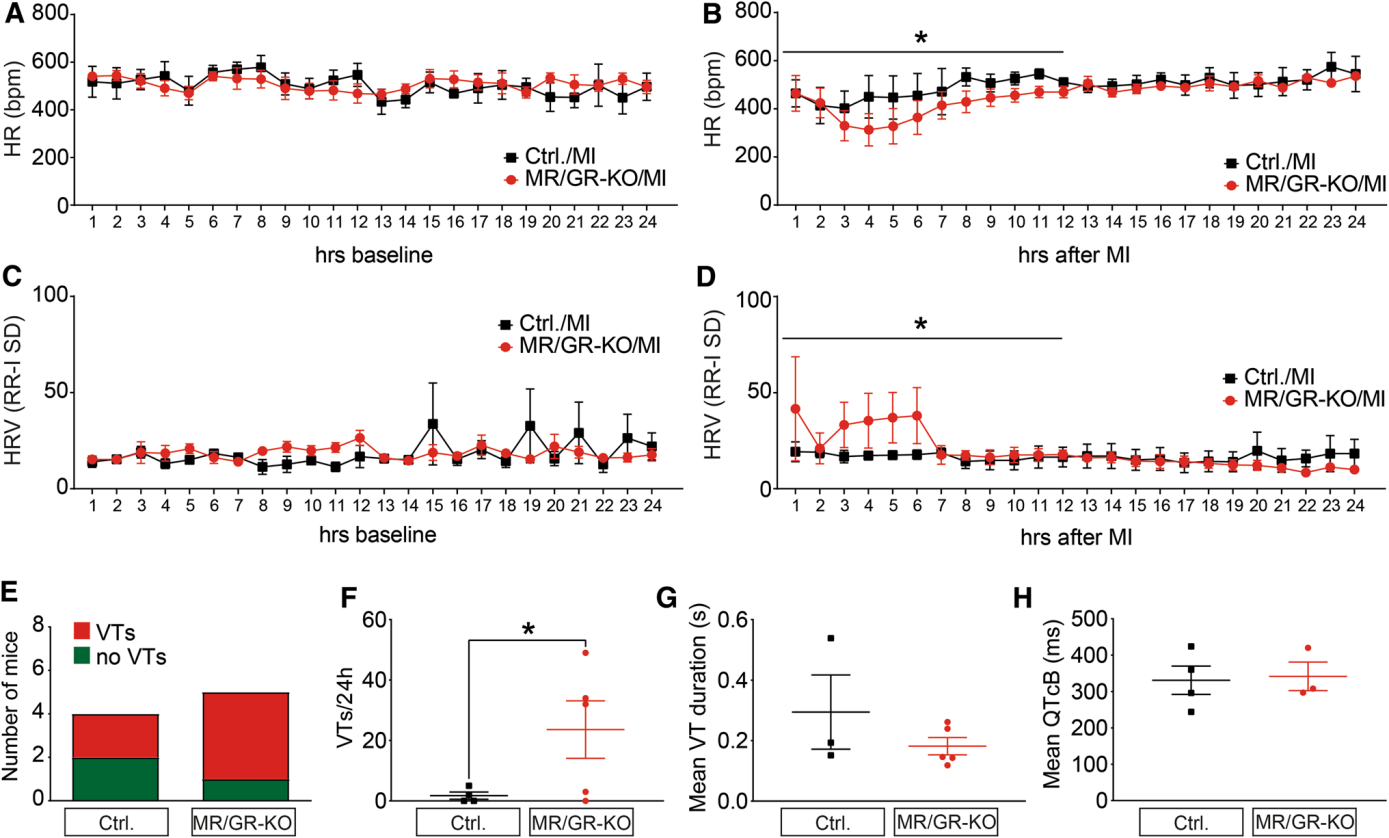
Ablation of the forebrain MR/GR blunts sympathetic activity and facilitates VTs after MI. At baseline MR/GR-KO mice did not show a significant phenotype with respect to heart rate (HR) or heart rate variability (HRV) in 24 h telemetry monitoring (*n* = 4–6/group) (**A**, **C**). KO mice show significant bradycardia with increased HRV during the first 12 h after MI when compared to Ctrl. mice (*n* = 3–6/group) (**B**, **D**) and suffer from a larger number of ventricular tachycardias (VTs) in the first 24 h (*n* = 3–6/group) (**E**–**G**) with unchanged Bazett-corrected QT interval duration (QTcB) (*n* = 3–4/group) (**H**). Data are presented as mean ± SEM, *n* = 3–6/group. *p < 0.05 by paired *t* test (**A**–**D**) or student’s *t* test (**E**–**H**)

At baseline, MR/GR-KO did not show a significant phenotype with respect to heart rate (HR) (Fig. 2A) or heart rate variability (HRV) in 24 h telemetry monitoring (Fig. 2C). In contrast, MR/GR-KO show significant bradycardia (Fig. 2B) with increased HRV during the first 12 h after MI (Fig. 2D) and suffer from a larger number of ventricular tachycardias (VTs) over the course of 24 h upon MI (Fig. 2E). Over the course of 3 days after MI, this effect was even more pronounced (Suppl. Figure 5B). Mean VT duration did not differ significantly between groups (Fig. 2G). We did not observe differences in Bazett-corrected QT interval duration (QTcB) between Ctrl. and MR/GR-KO mice (Fig. 2H).

### Ablation of the forebrain MR/GR causes HPA upregulation, catecholamine depletion and inhibits serine-16 phospholamban phosphorylation upon MI

**Fig. 3:**
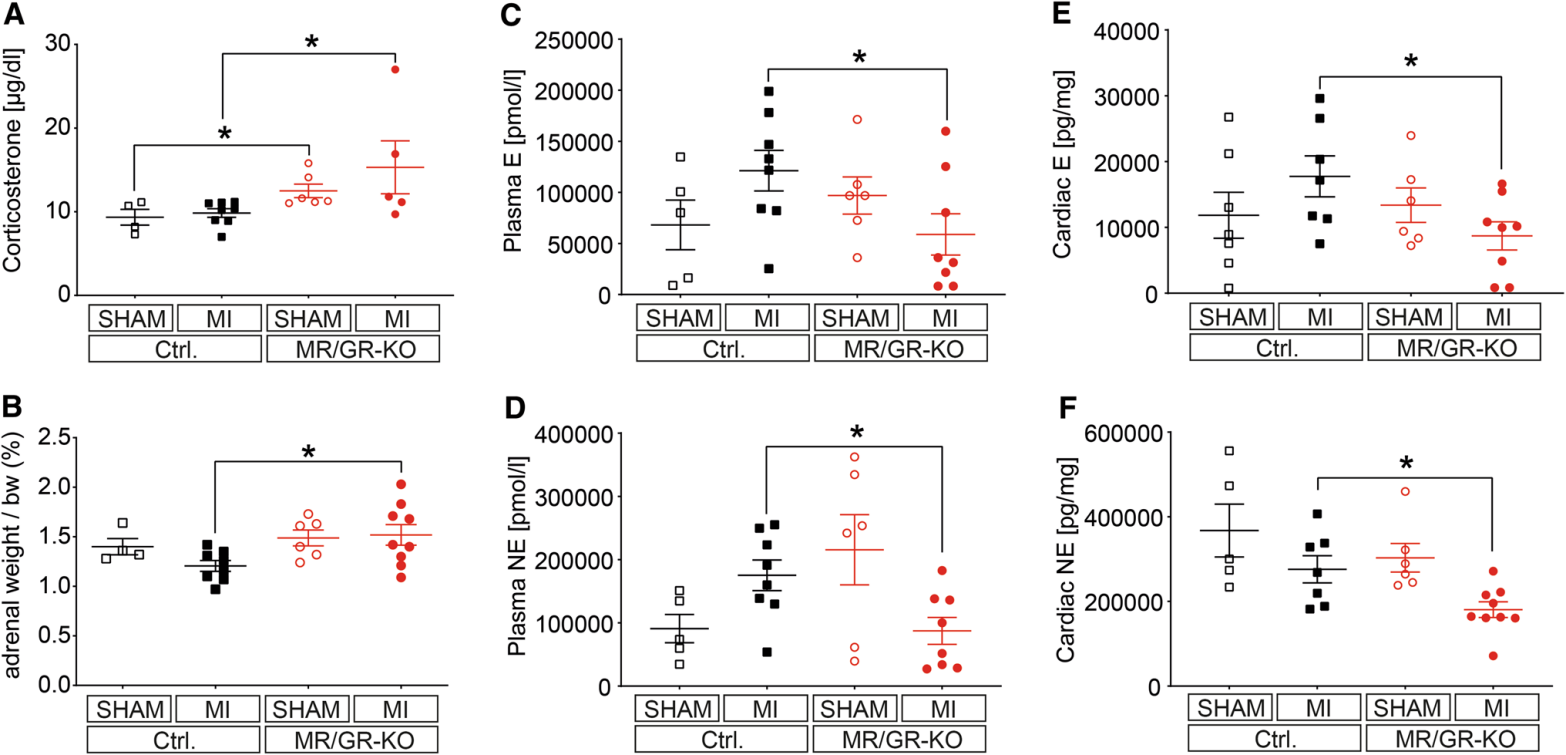
Ablation of the forebrain MR/GR causes HPA upregulation and catecholamine depletion. At 24 h after MI, MR/GR-KO mice display a significant increase of hypothalamo-pituitary-adrenal (HPA) axis activation, shown by elevation of serum corticosterone levels (*n* = 5–9/group) (**A**). Adrenal to body weight ratio supported this finding (*n* = 4–9/group) (**B**). Plasma epinephrine (E) (**C**) and norepinephrine (NE) (**D**) were sign. reduced upon MI in KO mice whereas there was a conflicting trend in control (Ctrl.) mice (*n* = 5–8/group). Also, cardiac left ventricular E and NE stores (**E**, **F**) were markedly blunted (*n* = 5–9/group) 24 h after MI. Data as mean ± SEM. *p < 0.05, ***p < 0.001 by ANOVA

**Fig. 4:**
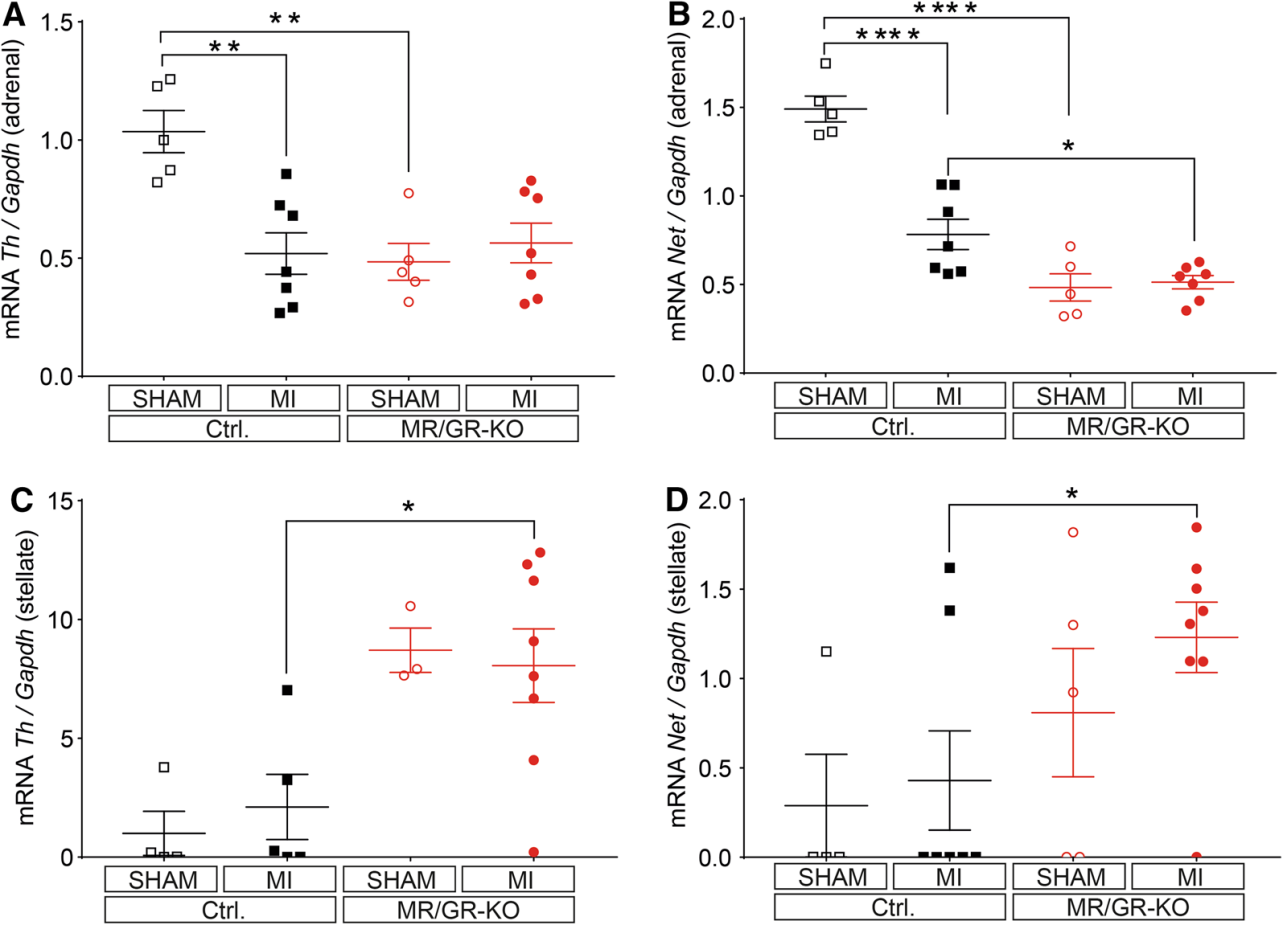
Ablation of the forebrain MR/GR divergently regulates adrenal and stellate ganglion catecholamine synthesis and reuptake. At 24 h, MR/GR-KO mice display blunted adrenal tyrosine hydroxylase (TH) (**A**) and norepinephrine transporter (NET) expression (**B**) compared to control (Ctrl.) mice after MI, with no significant changes inflicted by MI in KO mice (*n* = 5–7/group). TH (**C**) and NET expression (**D**) were upregulated in stellate ganglia of MR/GR-KO mice (*n* = 3–8/group). Data are presented as mean ± SEM. *p < 0.05, **p < 0.01, ***p < 0.001 by ANOVA

**Fig. 5:**
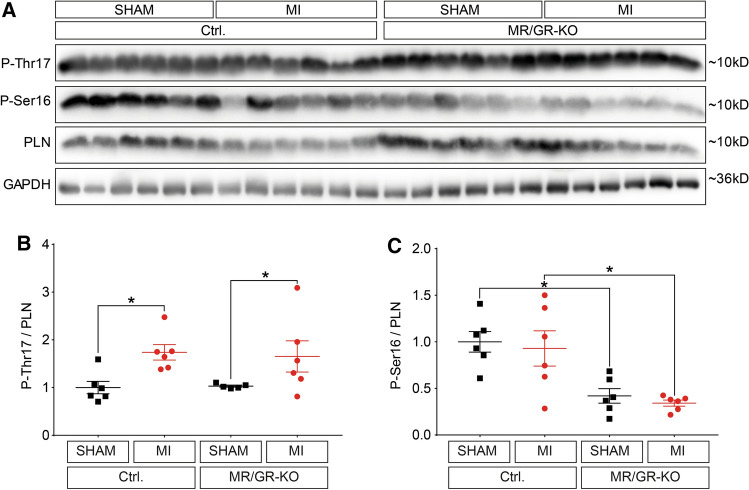
Ablation of the forebrain MR/GR reduces serine-16 phospholamban phosphorylation upon MI. Western blotting analysis of total phospholamban (PLN), phosphorylated Serine 16 (P-Ser16) and Threonine 17 (P-Thr17) from left ventricular samples 24 h after sham or MI operation (**A**) reveals unchanged Thr17 phosphorylation (**B**) and a reduction of Ser16 phosphorylation by more than 50% (**C**) in mice devoid of the forebrain MR and GR compared to control mice (Ctrl.). This finding suggests significantly reduced myocardial protein kinase A (PKA) activity in MR/GR-KO mice (*n* = 6/group). Data are presented as mean ± SEM. *p < 0.05 by ANOVA

The finding of increased depressive-like behavior in MR/GR-KO after MI (Fig. 1B) is mirrored by a significant increase of hypothalamo–pituitary–adrenal (HPA) axis activation, shown by elevation of serum corticosterone (Fig. 3A). We observed the lowest corticosterone levels (~ 2 µg/dl) in Ctrl. mice at resting state between 8 and 10AM 4 weeks after intervention, with additional marked elevation in MR/GR-KO mice, particularly after MI. In the setting of mild stress after echocardiography corticosterone levels increased in Ctrl. mice (~ 6 µg/dl), again with additional elevation in MR/GR-KO mice, particularly after MI. Consistently, the highest corticosterone levels in Ctrl. mice (~ 10 µg/dl) were observed 24 h after intervention and echocardiography, again with a significant increase in MR/GR-KO after MI. Adrenal to body weight ratio supported this finding (Fig. 3B). Plasma epinephrine (Fig. 3C) and norepinephrine (Fig. 3D) were markedly reduced upon MI in KO mice whereas there was a conflicting trend in Ctrl. mice. With respect to cardiac tissue catecholamines, left ventricular epinephrine (Fig. 3E) and norepinephrine stores (Fig. 3F) were markedly blunted. In KO mice adrenal tyrosine hydroxylase (TH) (Fig. 4A) and norepinephrine transporter (NET) expression (Fig. 4B) were blunted compared to Ctrl. mice after MI, with no significant changes inflicted by MI in MR/GR-KO. TH (Fig. 4C) and NET expression (Fig. 4D) were upregulated in stellate ganglia (Ggl.) of MR/GR-KO mice. Western blotting analysis of total phospholamban (PLN), phosphorylated Serine 16 (P-Ser16) and Threonine 17 (P-Thr17) PLN from left ventricular samples 24 h after sham or MI operation (Fig. 5A) reveals unchanged Thr17 phosphorylation (Fig. 5B) and a reduction of Ser16 phosphorylation by more than 50% (Fig. 5C) in mice devoid of the forebrain MR and GR. This finding suggests significantly reduced myocardial PKA activity in KO mice after myocardial infarction.

### Ablation of the forebrain MR/GR causes increased parasympathetic activity after MI

**Fig. 6:**
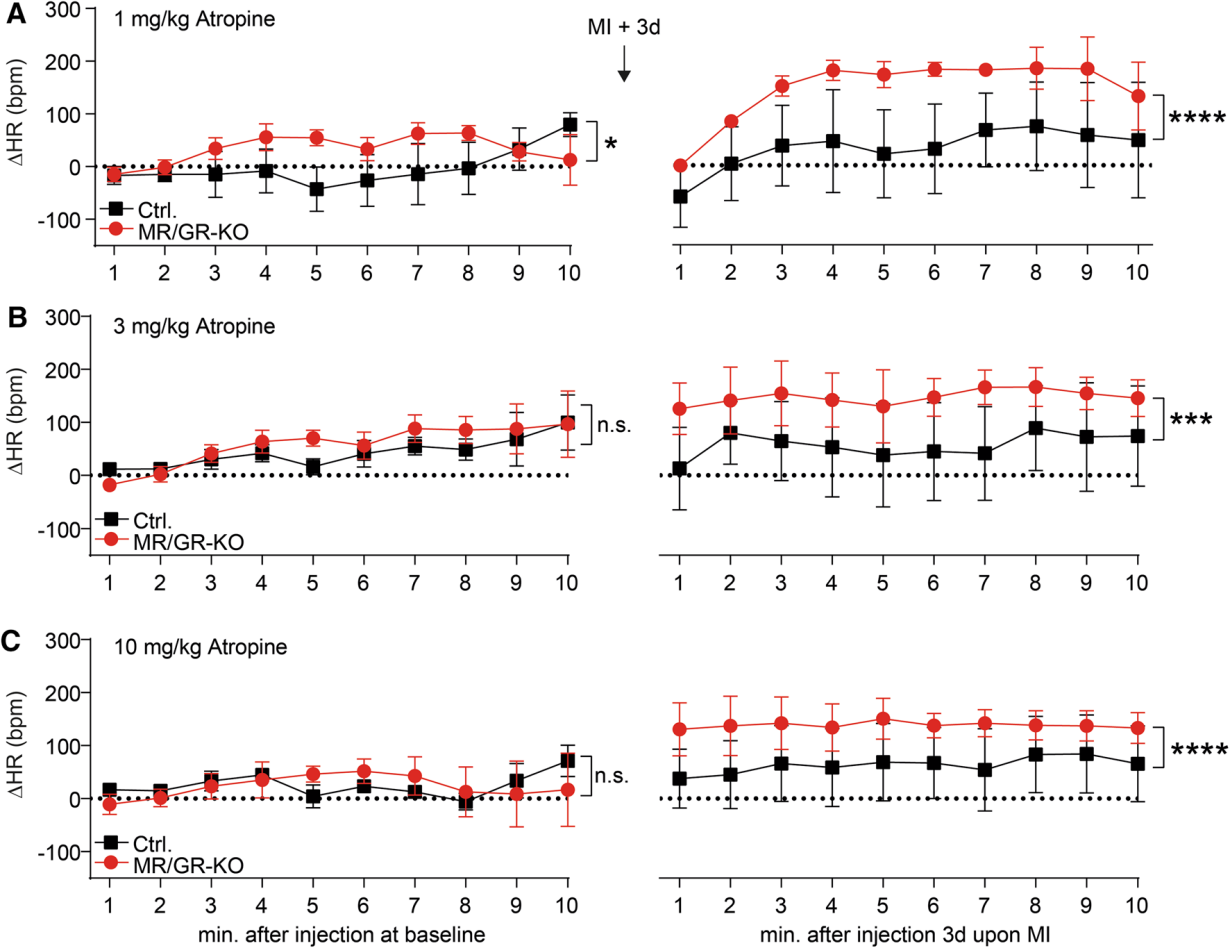
Ablation of the forebrain MR/GR causes increased parasympathetic activity after MI. Four days after MI, MR/GR-KO mice showed an elevated response to 1- (**A**), 3- (**B**), and 10 mg of atropine per kg (**C**) when compared to control (Ctrl.) mice. This effect was not present to this extend before myocardial infarction (*n* = 4–6/group). Data are presented as mean change compared to saline injection (∆) ± SEM. *p < 0.05 by paired *t* test

**Fig. 7:**
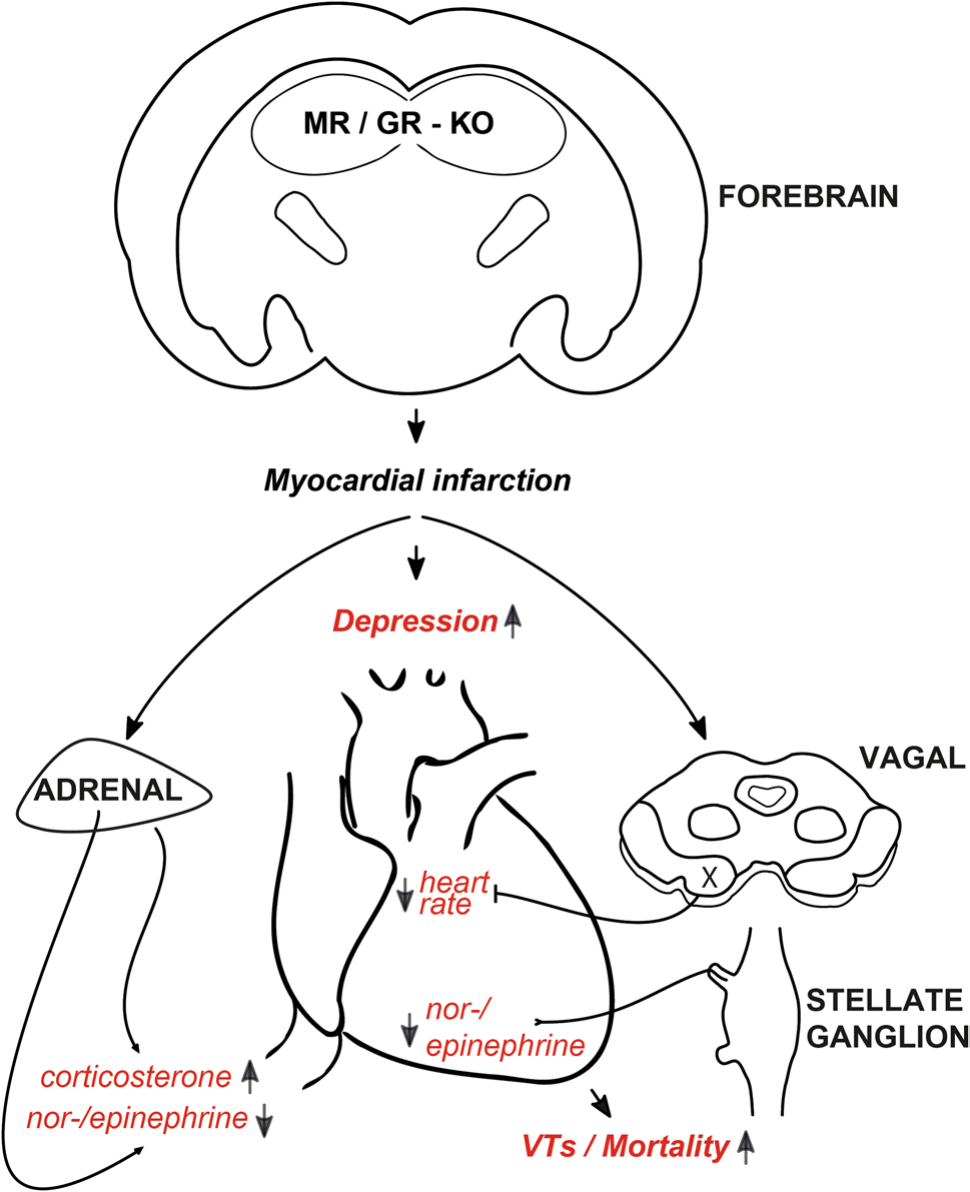
Forebrain MR/GR disequilibrium facilitates depression and increased mortality after MI. Ablation (KO) of limbic (forebrain) mineralo- and glucocorticoid receptors (MR/GR)—which have been associated with depressive states clinically—increase the experimental vulnerability toward stress-induced depression with subsequent sympathetic inhibition and increased parasympathetic (X) tone with systemic and cardiac catecholamine (nor-/epinephrine) depletion and elevated ventricular tachycardias (VTs). This finding may explain increased mortality of heart failure patients suffering from depression and the ineffectiveness of psycho- and pharmacological anti-depressive therapy regarding outcome

To test, whether an overactivation of the parasympathetic nervous system contributes to bradycardia, we conducted specific pharmacological interventions. Four days after MI, MR/GR-KO showed a significantly elevated heart rate response to 1- (Fig. 6A), 3- (Fig. 6B), and 10 mg of atropine per kg body weight (Fig. 6C) when compared to Ctrl. mice. This effect was not present to this extend before myocardial infarction. Taken together, we observed a significantly increased response to parasympathetic inhibition upon myocardial infarction in MR/GR-KO suggestive of sympathetic inhibition by parasympathetic overdrive (Fig. 7).

## Discussion

The CR hypothesis of depression postulates impaired central mineralocorticoid- (MR) and glucocorticoid receptor (GR) signaling as a key mechanism in the pathogenesis of human depression [28]. Disruption of the limbic corticosteroid receptor equilibrium has been proposed to cause failure of adequate behavioral adaptation to stress, resulting in depression after aversive events [38] such as MI. The potential causative contribution of forebrain CR signaling in the setting of MI or HF has, however, not been investigated. Here, we investigate the role of the CR hypothesis of depression and its impact on outcome after MI and HF.

Our study identified a pro-survival role of forebrain CRs upon MI. MR/GR-KO mice showed markedly increased mortality after MI with no change in left ventricular ejection fraction or myocardial damage and infarct size when compared to Ctrl. mice. MR/GR-KO also displayed significantly exacerbated depressive-like behavior after MI and enhanced activation of the HPA axis together with overall catecholamine depletion, while no endocrine phenotype was detectable at baseline in unstressed experimental animals. In line with reduced systemic and cardiac catecholamines, telemetry analysis revealed blunted sympathetic and increased parasympathetic activity with increased malignant arrhythmia in KO mice upon MI. In summary, our findings support the CR hypothesis of human depression in vivo and show that disrupted limbic CR signaling causes significant cardiac-related mortality after MI.

### Ablation of the forebrain MR/GR exacerbates depressive-like behavior after MI

MI significantly increased depressive-like behavior and serum corticosterone (CS) in MR/GR-KO mice when compared to Ctrl. mice, while not affecting overall motility or anxiety-like behavior. In line with this, we have recently shown that HF upon MI causes a predisposition toward depressive-like behavior but not necessarily depressive-like behavior in mice [9]. Other publications have suggested experimental MI and HF as a trigger of depressive-like but not anxiety-like behavior [17, 47, 57]. High CS was shown to facilitate HF-induced depressive-like behavior in mice [48] and inhibit neuronal differentiation of progenitor cells in the adult hippocampus [60]. Mice lacking the forebrain MR, displayed normal CS levels after stress [6] and were reported to show an unremarkable behavioral phenotype [54]. On the contrary, the GR has been implicated as a pivotal regulator of the HPA axis, causing CS downregulation by feedback inhibition via the pituitary, hypothalamus and hippocampus [10, 22, 29]. Two types of negative feedback to the HPA axis have been identified. The first is negative feedback of CS implemented at the level of the pituitary, which depends on the overall concentration of cortisol. The second process, so called fast feedback, depends on the rate of CS change, and involves interactions with hypothalamic and hippocampal GR receptors. Loss of fast CS feedback regulation has been described in the setting of depression [61]. Mice with a global 50% GR gene dose reduction displayed significant stress-induced depressive-like behavior with elevated CS levels immediately after immobilization stress [42]. An unconditional KO of the forebrain GR was reported to cause increased depressive-like behavior and HPA activity [49]. An inducible forebrain GR KO was shown to cause CS upregulation and depressive-like behavior in one study [7], whereas findings from Vogt et al. suggest otherwise with respect to behavior [54]. Here, we observed significant disruption of negative feedback in response to stress in mice devoid of the limbic MR and GR, which supports the concept that the imbalance of MR vs. GR causes HPA axis dysregulation [38, 52]. Therefore, our findings support the CR hypothesis of depression experimentally and add myocardial infarction as an extraordinary trigger of depressive-like behavior with HPA axis activation in the setting of impaired limbic CR signaling.

### Ablation of the forebrain MR/GR causes catecholamine depletion with bradycardia, ventricular tachycardia, and significant mortality upon MI

We observed blunted plasma and left ventricular catecholamines in KO mice upon MI with similar high-sensitive Troponin T levels at 24 h, suggestive of comparable infarct size with significant catecholamine depletion [59]. Also, left ventricular EF was similarly reduced 24 h, 2-, 4-, and 6 weeks after MI with no basal echocardiographic phenotype of KO mice. Telemetry analysis revealed marked bradycardia and increased heart rate variability in the first 12 h after MI, indicating increased parasympathetic drive in MR/GR-KO confirmed by atropine challenge.

However, there are several limitations of heart rate and HRV in assessing cardiac autonomic tone [23]. First, respiratory sinus arrhythmia (RSA) originates from the nucleus ambiguous, while negative chronotropy is triggered by the dorsal motor nucleus of the vagus. Second, respiratory parameters affect RSA and drive HRV independent of cardiac vagal activity [25]. Third, HRV represents the autonomic outflow targeting the sinoatrial pacemaker cells [24, 44]. Autonomic nervous function of other systems cannot be known from HRV but are suggested solely based on the idea of parallel regulation [23]. This of course is not always the case, since e. g. food intake enhances vagal activity to drive digestion but increases heart rate to meet digestive perfusion demand. Thus, to complement telemetry findings, we additionally analyzed systemic and left ventricular catecholamines as well as tyrosine hydroxylase expression in the stellate and adrenals as markers of autonomic regulation. System specific regulation is suggested by our TH expression data, which is upregulated in the stellate but blunted in the adrenals, indicative of increased cardiac but reduced systemic catecholamine demand. VT occurrence and mortality were significantly higher in MR/GR KO mice, suggestive of a causative role of vegetative disbalance.

Depleted cardiac norepinephrine with elevated plasma levels are a common finding in established HF [34], resembling sympathetic overdrive with subsequent elimination of cardio-sympathetic compensatory capacity. In this setting, impaired cardiac norepinephrine (NE) reuptake by neuronal NE transporter (NET) inhibition contributes to cardiac NE depletion [4]. Conversely, early after experimental coronary artery occlusion, epinephrine and norepinephrine were shown to be increased in the ischemic but not in non-ischemic myocardium [2, 35]. Since measurements were conducted from non-infarcted LV myocardium in our study, it is not surprising that we observed a non-significant increase of LV epinephrine as opposed to a non-significant decrease of norepinephrine as early as 24 h after MI in wildtype mice. In MR/GR-KO mice, we observed significantly blunted LV epinephrine and norepinephrine compared to control mice after MI, either indicative of cardiac SNS dominance or reduced central sympathetic outflow. Francis et al. have shown that intra-cerebroventricular MR antagonism reduces sympathetic drive in HF [13]. Loss of feedback HPA feedback regulation with high endogenous corticosterone—as observed in our study—may play a role here, which has shown the capacity to inhibit SNS activity [8, 36]. In our study, elevated cardio-sympathetic outflow is further supported by stellate TH expression data.

The observed increase of VTs in the setting of bradycardia after MI in MR/GR-KO mice appears contradictory at first and contrasts the traditional view that sympathetic activation is detrimental in myocardial ischemia [27], whereas vagal activation is beneficial [26]. However, the view of the sympathetic and parasympathetic inputs to the heart as polar antagonists is antiquated [19]. Rather they should be viewed like the concept of yin and yang [40], different but often complimentary with potential coactivation on a gradual scale. During simultaneous coactivation, principal response direction is determined by the dominating autonomic branch but allows more accurate and stable fine tuning of target organ function. In this regard, it has been shown that during simultaneous cardiac vago-sympathetic coactivation, vagal activity is mainly responsible for chronotropic effects, whereas sympathetic outflow rather targets the ventricular myocardium [31, 40]. Interestingly, simultaneous vagal and sympathetic cardiac signaling has been suggested to facilitate arrhythmogenesis. Thus, coactivation may play a role in the absence of the limbic MR/GR in our study, mirrored by myocardial catecholamine depletion and elevated stellate TH expression in combination with bradycardia. The latter is suggestive of selective cardiac LV catecholamine upregulation, which hints at a distinct regulation of sympathetic activity with respect to the LV as opposed to vagal regulation of chronotropy and might also explain an increased occurrence of VTs. However, the finding of reduced phosphorylation of PKA targets by western blotting in LVs suggests otherwise. Also, bradycardia as well as increased CS, both of which we have observed here, have been shown to potentially cause lethal ventricular arrhythmias [43, 45]. In this regard, the increased occurrence of VTs in MR/GR-KO mice upon MI reflects the clinical finding of an increased risk of sudden cardiac death (SCD) associated with depression [14, 16]. In line with this finding, a study utilizing the chronic mild stress model of depression showed increased susceptibility to ventricular arrhythmia, a known predictor of cardiac-related mortality/SCD [20]. We did not observe QT prolongation in MR/GR-KO compared to Ctrl. mice, suggestive of terminal arrhythmia rather than arrhythmogenic death in KO mice. With respect to our finding of unaffected infarct size and cardiac function, most patients who die of SCD after MI also do not suffer from a worse ejection fraction than the corresponding survivors [5]. However, Frey et al. observed significant depressive-like behavior triggered by MI to be correlated to infarct size, while no subgroup analysis regarding the impact of the behavioral phenotype on cardiac function was conducted [17]. Shi et al. have also shown increased susceptibility to ventricular arrhythmia in an animal model of depressive-like behavior after MI, based on sympathetic hyperactivation and exacerbated myocardial remodeling [47]. However, in this study, a blunted EF was observed as opposed to our findings here. Tao et al. have also observed a significant impact of a murine model of depression on cardiac function in myocardial ischemia–reperfusion injury (IR) [50]. The discrepant findings regarding infarct size and EF may be explicable by several differences. The aforementioned studies report an effect of different models of depression, i.e. chronic mild stress [47] or sleep deprivation [50], on ejection fraction as opposed to our study. Also, induction of depressive-like behavior occurred prior of myocardial injury—thus, potentially causing HPA/SNS axis disinhibition (first hit) before MI (second hit)—and therefore differ from our experimental approach, to investigate the effect of depression *caused by* MI in a setting of a limbic predisposition. In our setting, myocardial intervention triggers neuroendocrine and finally cardiac alterations, becoming detrimental only in the setting of HF due to MI. Moreover, these studies conducted sucrose preference testing for anhedonia, a different symptom of depression. In line with our finding of increased mortality in the absence of the forebrain MR/GR, a crucial role of the forebrain MR in survival of the acute phase of cardiac ischemia has been observed in rats [18]. Moreover, elevated CS levels are independently associated with mortality in patients suffering from HF [32].

In summary, the CR hypothesis of human depression may at least in part explain the increased mortality of patients suffering from depression after MI and the lack of beneficial impact on outcome of anti-depressive psycho- and pharmacotherapy. We propose SNS inhibition due to absence of the limbic MR with predominant HPA axis activation and impact on behavior by limbic GR deficit in response to MI to cause increased vulnerability to ventricular tachycardia and mortality upon MI. However, it remains unclear whether autonomic imbalance upon MI presents a temporary or a persistent change. Due to the beneficial impact of MR antagonists regarding cardiovascular morbidity/mortality after acute MI, and the local forebrain depletion of MR/GR in our study, one struggles to advise against MR (or GR) antagonism in the clinical setting of comorbid depression after MI. However, we suggest caution regarding the use of parasympathomimetic drugs in patients suffering from MI with comorbid depression. Further experimental studies need to assess the impact of aldosterone and GR antagonists, as well as Digitalis in the setting of ischemic heart disease and manifest depression. Our finding of acute divergent HPA and SNS regulation following MI needs to be clinically investigated and may present a screening tool to identify patients at high risk due to defective limbic CR signaling.

### Limitations

In this study, we used the model of permanent LAD ligation (PL) to induce MI. PL results in a larger infarct size with subsequent heart failure, since most of the area at risk is injured by long-term hypoxia and apoptosis compared to the model of myocardial ischemia/reperfusion (IR). IR mimics the setting of patients undergoing coronary intervention with smaller, more variable infarct size and often reduced heart failure due to initial partial hypoxic apoptosis followed by a second wave of necrosis of some of the area at risk [11]. We chose the model of PL, since we aimed to investigate the impact of large myocardial infarction (with clinical relevance to about 30% of MI patients due to very late reperfusion) and heart failure in the absence of forebrain MR/GR. However, this approach does not address the impact of reperfusion injury. Behavioral testing was conducted 4 weeks after intervention on 3 consecutive days. It has been shown that behavioral test batteries may impact rodent behavior [37] and we cannot rule out an impact of daily testing on the results. However, all mice underwent the same test protocol, which led us to disregard this possible confounder. Even though the Porsolt-Forced-Swim Test and Tail-Suspension Test present commonly used tests of depressive-like behavior [21] and are predictive models of antidepressant activity [1], transferability to human behavior needs to be regarded critically. All efforts were made to ensure validity of the results.

## Supplementary Information

Below is the link to the electronic supplementary material.

Supplementary Fig. 1 CaMKIIα-Cre driven ablation of the MR and GR in the forebrain. Expression of MR and GR mRNA in the prefrontal cortex (A, B), in the hippocampus (C, D), in the hypothalamus (E, F) and in the left ventricle (G, H). KO mice show significantly blunted corticosteroid receptor expression in the prefrontal cortex and the hippocampus, while no significant effect is observed in the hypothalamus or the left ventricle of the heart. Data were normalized to the mean of the Ctrl.-group and are presented as mean ± SEM. *P < 0.05 by student’s t test, n=9-10/group. (TIF 3252 KB)

Supplementary Fig. 2 Ablation of the forebrain MR/GR per se does not impact corticosterone or catecholamines. In MR/GR KO mice, serum levels of corticosterone (A), epinephrine (Plasma E) (C), norepinephrine (Plasma NE) (E) and dopamine (Plasma Dopa) (G) as well as left ventricular cardiac epinephrine (Cardiac E) (B), norepinephrine (Cardiac NE) (D) and Dopamine (Cardiac Dopa) (F) remain unchanged when compared to controls (Ctrl.) at baseline. Data are presented as mean ± SEM. *P < 0.05 by student’s t test, n=8-10/group. (TIF 3030 KB)

Supplementary Fig. 3 Ablation of the forebrain MR/GR does not impact infarct size, cardiac function, heart- and body weight after MI. At 24h, high-sensitive troponin T was similarly upregulated in plasma of control (Ctrl.) and KO mice after MI (A). Natriuretic peptide B (Nppb) was similarly affected by MI in Ctrl. and KO mice (n=5-6/group) (B). Heart weight/tibia length measurements confirmed the previous results, showing no significant alteration between Ctrl. and KO after MI (n=12-18/group) (C). Similar body weight at baseline and after myocardial infarction in Ctrl. and KO mice (D). Data are presented as mean ± SEM. *P < 0.05 by ANOVA. (TIF 2874 KB)

Supplementary Fig. 4 Ablation of the forebrain MR/GR exacerbates depressive-like but not anxiety-like behavior after myocardial infarction (MI). At 4 weeks, the Open field test revealed unaffected total distance moved (A), mean distance to center (B) and speed (C) (n=6-8/group). In the Porsolt Forced-Swim test depressive-like behavior was confirmed (D) (n=4-7/group). MR/GR-KO mice were immobile significantly longer (%) when compared to Ctrl. mice after MI. The finding of increased depressive-like behavior in MR/GR-KO mice after MI were mirrored by a significant increase of hypothalamo-pituitary-adrenal (HPA) axis activation, shown here by elevation of serum corticosterone levels from resting (E) and mildly stressed mice after awake echocardiography (F) (n=5-9/group). Cardiac norepinephrine (Cardiac NE) (G) and epinephrine (Cardiac E) 4 weeks after intervention (H) (n=4-8/group). Mean ± SEM. *P < 0.05, **P < 0.01, ***P < 0.001 by ANOVA. (TIF 4534 KB)

Supplementary Fig. 5 Ablation of the forebrain MR/GR facilitates VTs 3d upon MI**.** A similar amount of control (Ctrl.) and KO mice suffer from VTs after MI (A). MR/GR KO mice display a significantly larger amount of ventricular tachycardias (VTs) within the first 3d after MI (n=6-7/group) (B). Total VT duration and mean VT duration did not differ significantly between groups (C, D). Data are presented as mean ± SEM. *P < 0.05 by student’s t test. (TIF 2876 KB)
