# Supplementary figures and images for: Forebrain corticosteroid receptors promote post-myocardial infarction depression and mortality
Source: Basic Res Cardiol. 2022 Sep 6;117(1):44. doi: 10.1007/s00395-022-00951-6 (PMC9448693; doi:10.1007/s00395-022-00951-6)

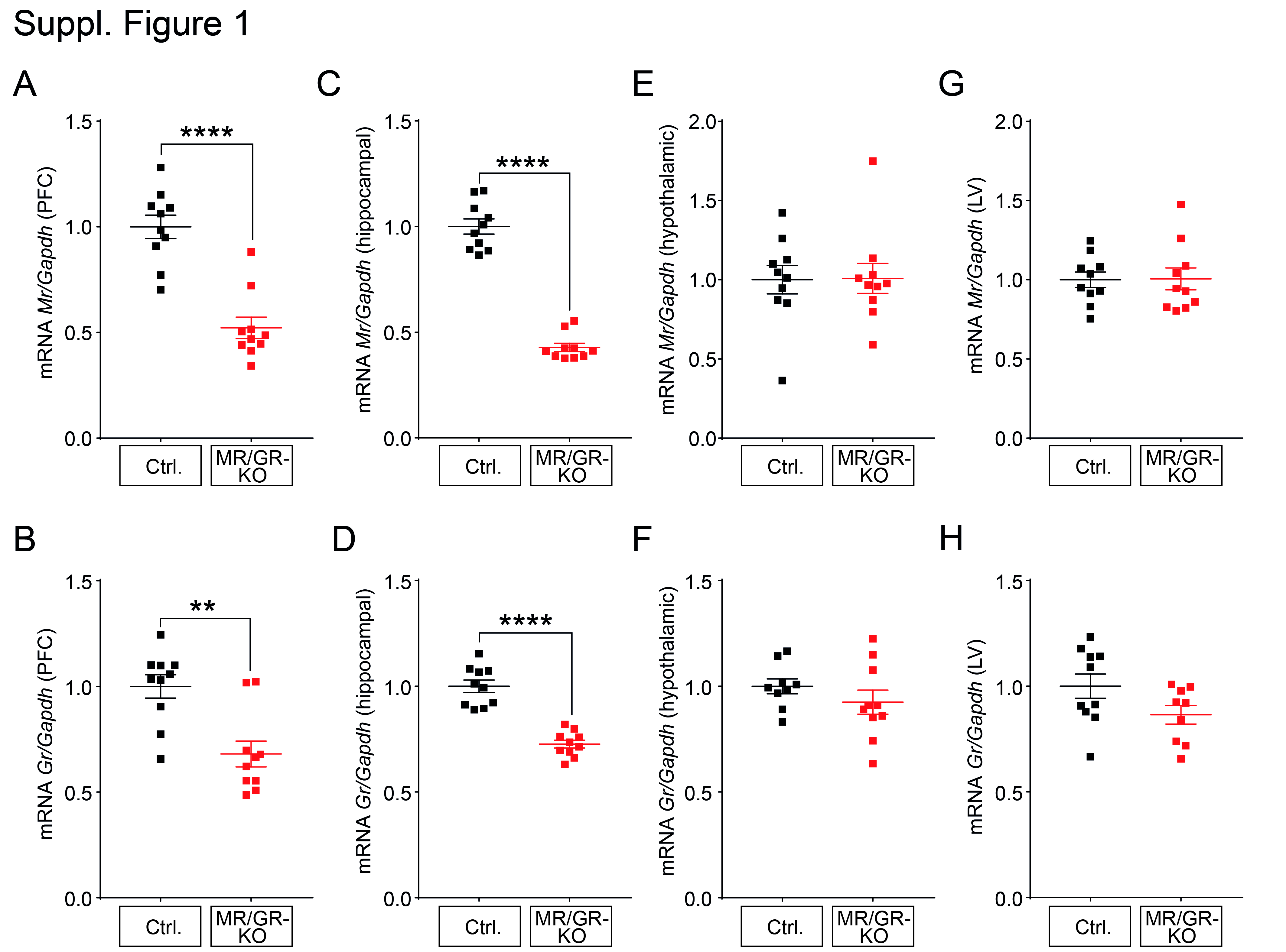

Supplement: Supplementary file 1 — Supplementary Fig. 1 CaMKIIα-Cre driven ablation of the MR and GR in the forebrain. Expression of MR and GR mRNA in the prefrontal cortex (A, B), in the hippocampus (C, D), in the hypothalamus (E, F) and in the left ventricle (G, H). KO mice show significantly blunted corticosteroid receptor expression in the prefrontal cortex and the hippocampus, while no significant effect is observed in the hypothalamus or the left ventricle of the heart. Data were normalized to the mean of the Ctrl.-group and are presented as mean ± SEM. *P < 0.05 by student’s t test, n=9-10/group. (TIF 3252 KB) [file 395_2022_951_MOESM1_ESM.tif]

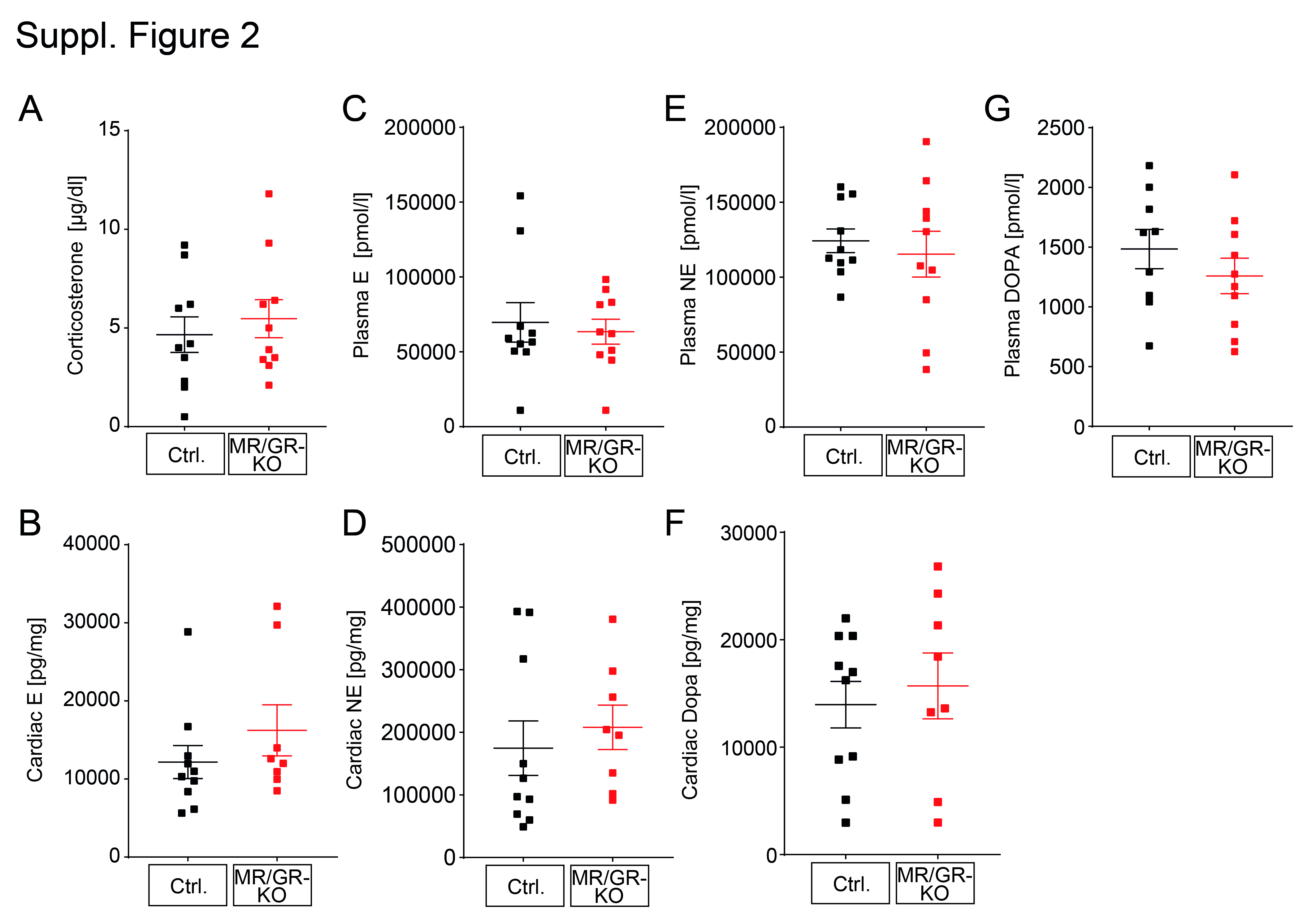

Supplement: Supplementary file 2 — Supplementary Fig. 2 Ablation of the forebrain MR/GR per se does not impact corticosterone or catecholamines. In MR/GR KO mice, serum levels of corticosterone (A), epinephrine (Plasma E) (C), norepinephrine (Plasma NE) (E) and dopamine (Plasma Dopa) (G) as well as left ventricular cardiac epinephrine (Cardiac E) (B), norepinephrine (Cardiac NE) (D) and Dopamine (Cardiac Dopa) (F) remain unchanged when compared to controls (Ctrl.) at baseline. Data are presented as mean ± SEM. *P < 0.05 by student’s t test, n=8-10/group. (TIF 3030 KB) [file 395_2022_951_MOESM2_ESM.tif]

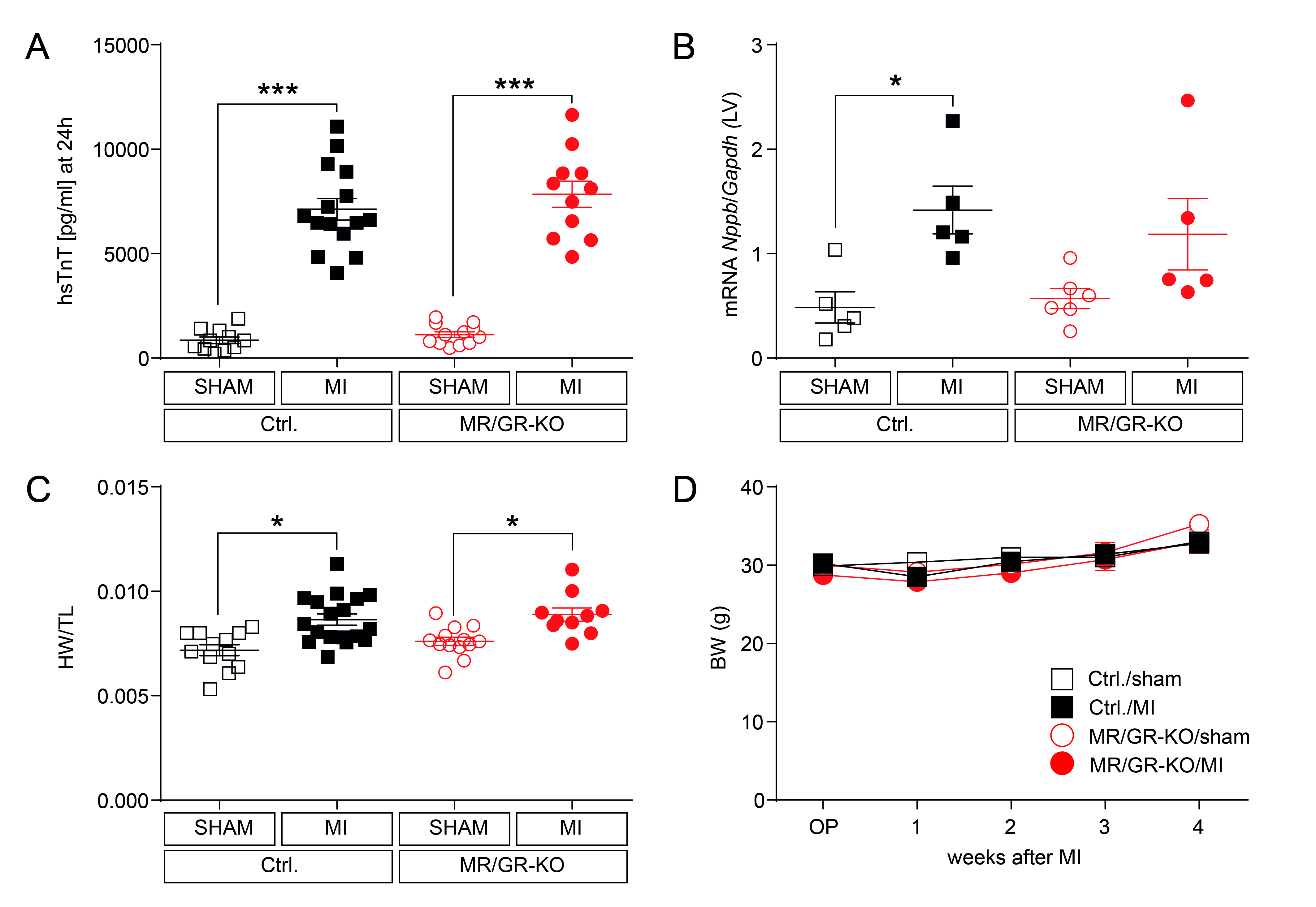

Supplement: Supplementary file 3 — Supplementary Fig. 3 Ablation of the forebrain MR/GR does not impact infarct size, cardiac function, heart- and body weight after MI. At 24h, high-sensitive troponin T was similarly upregulated in plasma of control (Ctrl.) and KO mice after MI (A). Natriuretic peptide B (Nppb) was similarly affected by MI in Ctrl. and KO mice (n=5-6/group) (B). Heart weight/tibia length measurements confirmed the previous results, showing no significant alteration between Ctrl. and KO after MI (n=12-18/group) (C). Similar body weight at baseline and after myocardial infarction in Ctrl. and KO mice (D). Data are presented as mean ± SEM. *P < 0.05 by ANOVA. (TIF 2874 KB) [file 395_2022_951_MOESM3_ESM.tif]

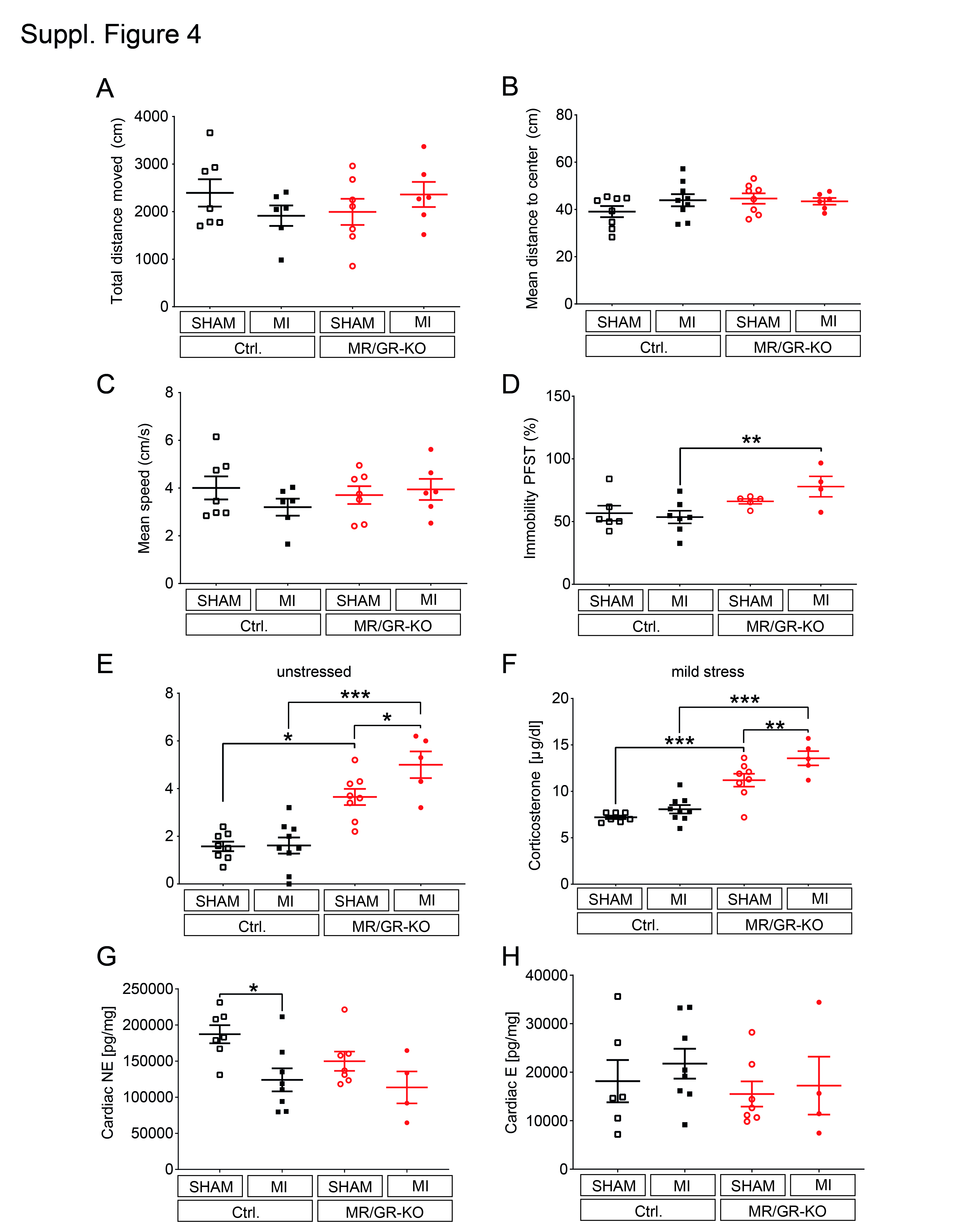

Supplement: Supplementary file 4 — Supplementary Fig. 4 Ablation of the forebrain MR/GR exacerbates depressive-like but not anxiety-like behavior after myocardial infarction (MI). At 4 weeks, the Open field test revealed unaffected total distance moved (A), mean distance to center (B) and speed (C) (n=6-8/group). In the Porsolt Forced-Swim test depressive-like behavior was confirmed (D) (n=4-7/group). MR/GR-KO mice were immobile significantly longer (%) when compared to Ctrl. mice after MI. The finding of increased depressive-like behavior in MR/GR-KO mice after MI were mirrored by a significant increase of hypothalamo-pituitary-adrenal (HPA) axis activation, shown here by elevation of serum corticosterone levels from resting (E) and mildly stressed mice after awake echocardiography (F) (n=5-9/group). Cardiac norepinephrine (Cardiac NE) (G) and epinephrine (Cardiac E) 4 weeks after intervention (H) (n=4-8/group). Mean ± SEM. *P < 0.05, **P < 0.01, ***P < 0.001 by ANOVA. (TIF 4534 KB) [file 395_2022_951_MOESM4_ESM.tif]

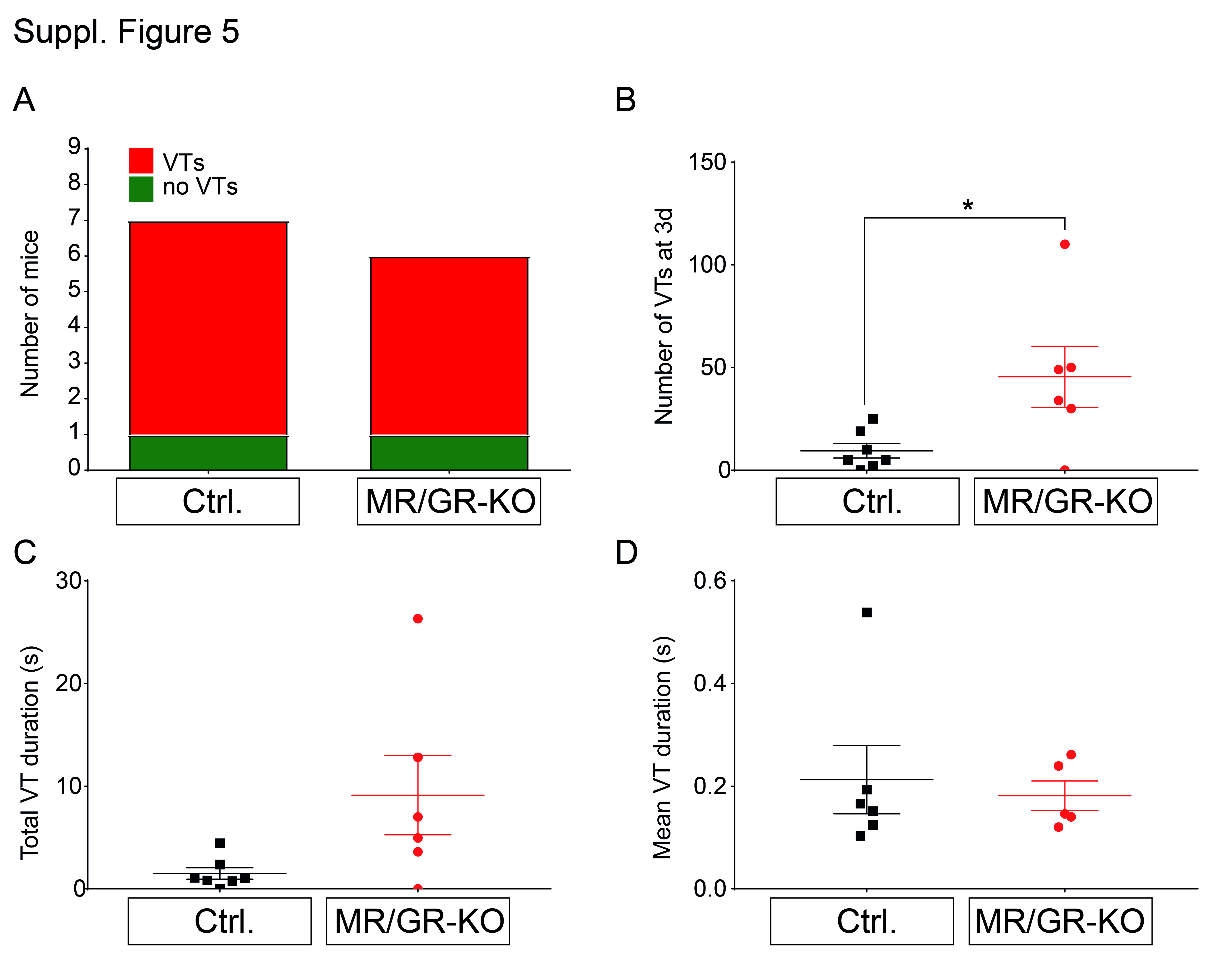

Supplement: Supplementary file 5 — Supplementary Fig. 5 Ablation of the forebrain MR/GR facilitates VTs 3d upon MI. A similar amount of control (Ctrl.) and KO mice suffer from VTs after MI (A). MR/GR KO mice display a significantly larger amount of ventricular tachycardias (VTs) within the first 3d after MI (n=6-7/group) (B). Total VT duration and mean VT duration did not differ significantly between groups (C, D). Data are presented as mean ± SEM. *P < 0.05 by student’s t test. (TIF 2876 KB) [file 395_2022_951_MOESM5_ESM.tif]
